# Supplementary material for: Effects of a Multidimensional Exercise and Mindfulness Approach Targeting Physical, Psychological, and Functional Outcomes: Protocol for the BACKFIT Randomized Controlled Trial with an Active Control Group
Source: Healthcare (Basel). 2025 Aug 20;13(16):2065. doi: 10.3390/healthcare13162065 (PMC12386025; doi:10.3390/healthcare13162065)
Supplement: Supplementary file 1 [file healthcare-13-02065-s001.zip › Supplementary File S2 BACKFIT TIDIER template.pdf]

The TIDieR (Template for Intervention Description and Replication) Checklist*:

Information to include when describing an intervention and the location of the information

Item number	Item	Where located **	
		Primary paper (page or appendi number)	Other † (details)
1.	BRIEF NAME<br>Provide the name or a phrase that describes the intervention.	Page 1	Effects of a Multidimensional Exercise and Mindfulness Approach Targeting Physical, Psychological, and Functional Outcomes: Protocol for the BACKFIT Randomized Controlled Trial with an Active Control Group
			However, few studies have explored multidimensional interventions that combine supervised exercise and mindfulness, particularly in a face-to-face format. The present clinical trial aims to contribute to this emerging fie by evaluating the effects of a structured, in-person intervention on a range of physical and psychological outcomes in individuals with CPLBP.<br>With the aim of understanding the effects and magnitude these multidimensional programs on health outcomes in individuals with CPLBP, the BACKFIT randomized controlled trial was initiated. The main objective is to determine the effectiveness of a multidimensional prograr

			(combining supervised physical exercise and mindfulness compared to supervised physical exercise alone and an active control group (usual care), on pain and disability (primary outcomes), body composition, muscular fitness, gait parameters, device-measured PA and sedentary behaviour, self-reported sedentary behaviour, quality of lif pain catastrophizing, mental health, sleep duration and quality, and central sensitization (secondary outcomes) in individuals with CPLBP.
2.	WHY<br>Describe any rationale, theory, or goal of the elements essential to the intervention.	Page 4	
	WHAT	Page11	This progression will be structured according to the following phases [32]. (i) Phase 1 (3 sessions, ≥5 RPE): mobility, isometric, and motor control exercises that will include full body, with special emphasis on the core, particularly isometric contraction of the transversus abdominis muscle. Participants will be instructed to place their hands on the lower abdomen during the exercises to perceive the contraction through kinesthetic feedback and facilitate correct transversus abdominis activation (ii) Phase 2 (4 sessions, ≥5 RPE): co-contraction and functional tasks involving deep trunk muscles. (iii) Phase 3 (4 sessions, ≥6 RPE): functional

			task with greater difficulty or intensity, including load.<br>(iv) Phase 4 (5 sessions, ≥6 RPE): functional task performed on an unstable surface.<br>A detailed description of the exercise intervention is shown in Tables 3 and 4.
3.	Materials: Describe any physical or informational materials used in the intervention, including those provided to participants or used in intervention delivery or in training of intervention providers. Provide information on where the materials can be accessed (e.g. online appendix, URL).	Page 17	A detailed description of the exercise intervention program is shown in tables 3 and 4.<br>Each session will include a topic presentation, group dialogue and exploration (using appreciative inquiry), and a mindfulness practice. Participants will receive workbooks, guided meditation audios, and instructions for home practice. Details are provided in Table 5.
4.	Procedures: Describe each of the procedures, activities, and/or processes used in the intervention, including any enabling or support activities.	Pages 12-20	Description of backfit protocol intervention. Tables 3, 4 and 5.
5.	WHO PROVIDED<br>For each category of intervention provider (e.g. psychologist, nursing assistant), describe their expertise, background and any specific training given.	Page 10	The physical exercise program will be supervised and guided, and all sessions will be conducted under the direct supervision of licensed (MD and Ph.D.) physical therapists and sports sciences specialists present physically. Mindfulness sessions will be taught by a professional accredited by Brown University who will not participate in any assessment phase. All the

			professionals will have experience working with this population.
6.	HOW<br>Describe the modes of delivery (e.g. face-to-face or by some other mechanism, such as internet or telephone) of the intervention and whether it was provided individually or in a group.	Page 10	The intervention program will be conducted face-to-face in groups at the Physical Medicine and Rehabilitation Service.
7.	WHERE<br>Describe the type(s) of location(s) where the intervention occurred, including any necessary infrastructure or relevant features.	Page 9-10<br><br>Page 10	On the first evaluation day, participants will report to the Multidisciplinary Laboratory in the Faculty of Sport Sciences (Granada) and will complete the following assessments: clinical information survey, blood pressure, resting heart rate, body composition, pain threshold, physical fitness, and gait parameters.<br><br>The intervention program will be conducted face-to-face in groups at the Physical Medicine and Rehabilitation Service.
	WHEN and HOW MUCH	Pages 10-11	The IG1 (supervised exercise program) will undergo an exercise program twice per week (45 min per session) for 8 weeks. Each session will include a warm-up (5

		min), muscle-strengthening exercises (35 min), and a cool-down (5 min, stretching exercises). The program will focus on core muscles, starting with low-intensity isometric contraction for trunk stabilization and mobility exercises, then increasing intensity with functional tasks. Exercise intensity will be moderate-to-vigorous, assessed via the RPE scale [43], ensuring no pain is felt. This scale will be visible to participants during sessions. Each exercise will last 60 seconds, followed by 60 seconds rest. Execution speed will be individualized to each participant's physical condition and tolerance. Exercises will follow a fixed order with progressive difficulty, as described in previous literature [32,44].
	Page 17	The IG2 (multidimensional program) will follow the same exercise protocol as IG1 (twice per week, 45 minutes per session) and also participate in a mindfulness program once per week (2.5 hours per session) for 8 weeks. The MBSR program will follow Jon Kabat-Zinn's protocol [45]. Each session will include a topic presentation, group dialogue and exploration (using appreciative inquiry), and a mindfulness practice. Participants will receive workbooks, guided meditation

			audios, and instructions for home practice. Details are provided in Table 5.
8.	Describe the number of times the intervention was delivered and over what period of time including the number of sessions, their schedule, and their duration, intensity or dose.	Page 17	The CG will receive the usual care provided by the Physical Medicine and Rehabilitation Service. Participants will follow the same session structure described above twice per week (45 minutes per session) for 8 weeks. All participants will perform 1 set of 10 repetitions or 60 seconds per exercise, followed by 60 seconds of rest. Execution speed will be individualized. Intensity will be assessed with an RPE scale (≥5 RPE).
9.	TAILORING<br>If the intervention was planned to be personalised, titrated or adapted, then describe what, why, when, and how.	N/A	
	MODIFICATIONS	Page 17	This program allows adaptations for practical evidence-based aspects, such as performing an alternative exercise targeting the same muscle group without causing pain or changing the plane of movement. Adverse events, effects, or health issues attributable to the testing or intervention sessions, along with their severity and resolution, will be recorded by a researcher responsible for auditing the assessment team. If an

10.‡	If the intervention was modified during the course of the study, describe the changes (what, why, when, and how).		adverse event prevents participation, temporary or permanent exclusion will be discussed and documented. Temporary exclusions require medical clearance for return.
11.	HOW WELL<br>Planned: If intervention adherence or fidelity was assessed, describe how and by whom, and if any strategies were used to maintain or improve fidelity, describe them.	Page 11	Participants will receive real-time feedback on posture correction and examples for exercise familiarization. Strategies to improve exercise adherence will include: (i) a positive and dynamic session environment; (ii) an attendance control system to minimize dropouts.
12.‡	Actual: If intervention adherence or fidelity was assessed, describe the extent to which the intervention was delivered as planned.	N/A	Not applicable

** **Authors** - use N/A if an item is not applicable for the intervention being described. **Reviewers** – use '?' if information about the element is not reported/not sufficiently reported.

† If the information is not provided in the primary paper, give details of where this information is available. This may include locations such as a published protocol or other published papers (provide citation details) or a website (provide the URL).

‡ If completing the TIDieR checklist for a protocol, these items are not relevant to the protocol and cannot be described until the study is complete.

* We strongly recommend using this checklist in conjunction with the TIDieR guide (see *BMJ* 2014;348:g1687) which contains an explanation and elaboration for each item.

* The focus of TIDieR is on reporting details of the intervention elements (and where relevant, comparison elements) of a study. Other elements and methodological features of studies are covered by other reporting statements and checklists and have not been duplicated as part of the TIDieR checklist. When a **randomised trial** is being reported, the TIDieR checklist should be used in conjunction with the CONSORT statement (see www.consort-statement.org) as an extension of **Item 5 of the CONSORT 2010 Statement.** When a **clinical trial protocol** is being reported, the TIDieR checklist should be used in conjunction with the SPIRIT statement as an extension of **Item 11 of the SPIRIT 2013 Statement** (see www.spirit-statement.org). For alternate study designs, TIDieR can be used in conjunction with the appropriate checklist for that study design (see www.equator-network.org).
